# Efficient Biodegradation of the Neonicotinoid Insecticide Flonicamid by *Pseudaminobacter salicylatoxidans* CGMCC 1.17248: Kinetics, Pathways, and Enzyme Properties

**DOI:** 10.3390/microorganisms12061063

**Published:** 2024-05-24

**Authors:** Yun-Xiu Zhao, Jing Yuan, Ke-Wei Song, Chi-Jie Yin, Li-Wen Chen, Kun-Yan Yang, Ju Yang, Yi-Jun Dai

**Affiliations:** 1 Jiangsu Key Laboratory for Bioresources of Saline Soils, Jiangsu Synthetic Innovation Center for Coastal Bio-Agriculture, School of Wetlands, Yancheng Teachers University, Yancheng 224007, China; zhaoyx@yctu.edu.cn (Y.-X.Z.); 13773187821@163.com (K.-W.S.); yinchijie@outlook.com (C.-J.Y.); 2 Jiangsu Key Laboratory for Microbes and Functional Genomics, Jiangsu Engineering and Technology Research Center for Industrialization of Microbial Resources, College of Life Science, Nanjing Normal University, Nanjing 210023, China; 231201019@njnu.edu.cn; 3 College of Marine and Biological Engineering, Yancheng Teachers University, Yancheng 224002, China; clw2212410431@163.com (L.-W.C.); carrie041029@outlook.com (K.-Y.Y.)

**Keywords:** biodegradation, *Pseudaminobacter salicylatoxidans*, amidase

## Abstract

Nitrile-containing insecticides can be converted into their amide derivatives by *Pseudaminobacter salicylatoxidans*. *N*-(4-trifluoromethylnicotinoyl) glycinamide (TFNG-AM) is converted to 4-(trifluoromethyl) nicotinoyl glycine (TFNG) using nitrile hydratase/amidase. However, the amidase that catalyzes this bioconversion has not yet been fully elucidated. In this study, it was discovered that flonicamid (FLO) is degraded by *P. salicylatoxidans* into the acid metabolite TFNG via the intermediate TFNG-AM. A half-life of 18.7 h was observed for *P. salicylatoxidans* resting cells, which transformed 82.8% of the available FLO in 48 h. The resulting amide metabolite, TFNG-AM, was almost all converted to TFNG within 19 d. A novel amidase-encoding gene was cloned and overexpressed in *Escherichia coli*. The enzyme, PmsiA, hydrolyzed TFNG-AM to TFNG. Despite being categorized as a member of the amidase signature enzyme superfamily, PsmiA only shares 20–30% identity with the 14 previously identified members of this family, indicating that PsmiA represents a novel class of enzyme. Homology structural modeling and molecular docking analyses suggested that key residues Glu247 and Met242 may significantly impact the catalytic activity of PsmiA. This study contributes to our understanding of the biodegradation process of nitrile-containing insecticides and the relationship between the structure and function of metabolic enzymes.

## 1. Introduction

The insecticide flonicamid (*N*-cyanomethyl-4-trifluoromethylnicotinamide, FLO) is a new systemic insecticide with selective action that is widely used for the treatment of aphids and whiteflies [1]. In harvested crops (e.g., cabbage, bell peppers, dried hops, cucumbers, and oranges) and water sources, the main metabolites of FLO, *N*-(4-trifluoromethylnicotinoyl) glycinamide (TFNG-AM), 4-(trifluoromethyl) nicotinoyl glycine (TFNG), and 5-trifluoromethylnicotinic acid (TFNA) have been detected [2,3,4,5]. In addition to this, traces of FLO have been found in human urine and serum samples [6,7]. The mouse genome can be significantly damaged by high doses of FLO [8]. FLO and its metabolites are highly soluble in water and persistent in the environment, which allow them to accumulate in humans [8,9]. Some scholars have attempted photo-oxidation-based remediation methods to address the residues of FLO in the environment. For example, when adding persulfate to test wastewater, the removal effect of FLO is not ideal. Although adding ZnO- and TiO_2_-coated magnetic particles to the system can significantly improve the degradation rate of FLO, it is not economically feasible [10,11,12]. With the increasing use of FLO and the increasing possibility of accumulation of FLO and its metabolites in the environment, adequate attention is needed.

Microbial remediation is a cost-effective, environmentally friendly technique that may remove residual pesticides or insecticides from soil and water [13,14]. Two degradation pathways—via nitrile hydratase (NHase)/amidase and nitrilase, respectively—are used by microbes to break down FLO [15]. According to our earlier research, the N_2_-fixing bacteria *Variovorax boronicumulans* CGMCC 4969, *Microvirga flocculans* CGMCC 1.16731, *Aminobacter* sp. CGMCC 1.17253, *Ensifer meliloti* CGMCC 7333, and *E. adhaerens* CGMCC 6315 efficiently convert FLO to TFNG-AM using NHases [16,17,18,19,20]. *V. boronicumulans* CGMCC 4969 and *M. flocculans* CGMCC 1.16731 hydrolyze TFNG-AM to TFNG using amidases but do not hydrolyze TFNA-AM, another intermediate of FLO. *Pseudomonas stutzeri* CGMCC 22915 effectively degraded TFNA-AM to 5-trifluoromethylnicotinic acid (TFNA) by using the amidase PsAmiA, but it could not degrade TFNG-AM [21]. Yang et al. [22] reported that a bifunctional nitrilase from *Alcaligenes faecalis* CGMCC 17553 degraded FLO into its amide products (TFNG-AM) and acids (TFNG). However, the enzymes responsible for this degradation are still unknown.

Nitrile-converting bacteria usually possess nitrilase and NHase/amidase systems [23]. The NHases (EC 4.2.1.84) are metalloenzymes that catalyze nitrile compounds into the corresponding amides. They are widely used as biocatalysts in the synthesis of fine chemicals, amino acids, and derivatives, and they play a crucial role in the degradation of toxic nitrile compounds [24,25]. As a class of hydrolases, amideases (EC 3.5.1.4) play a vital role in the biosynthesis of amide products and the elimination of toxic environmental pollutants, primarily by breaking the C–N bond of amides and catalyzing their hydrolysis to the corresponding carboxylic acid and ammonia [26]. The substrate spectrum of amidase signature family amidases has been widely studied, and all of them showed broad substrate specificity [27]. Amidases and NHases are co-expressed in the same gene cluster in wild-type microorganisms, and hence nitrile-containing compounds are ultimately metabolized to carboxylic acids [26,28]. *Variovorax boronicumulans*, *Agrobacterium*, *Rhizobium*, *Ensifer*, and *Bradyrhizobium* convert indole-3-acetonitrile (IAN) to indole-3-acetamide (IAM) via NHase and then IAM to indole-3-acetic acid (IAA) via amidase [29,30,31]. The NHase/amidase cascade enzymatic system can degrade nitrile compounds into less toxic acids [32].

In this study, we demonstrate that *Pseudaminobacter salicylatoxidans* CGMCC 1.17248 degrades FLO via the NHase/amidase enzymatic cascade system to produce TFNG-AM and TFNG. An investigation of the kinetics of TFNG-AM degradation by *P. salicylatoxidans* CGMCC 1.17248 was performed, and the amidase gene responsible for the conversion was cloned, overexpressed, and purified. This research improves our comprehension of the mechanisms of microbial FLO degradation. *P. salicylatoxidans* CGMCC 1.17248 has the potential to remediate nitrile-containing pesticide contamination.

## 2. Materials and Methods

### 2.1. Chemicals and Media

FLO and TFNG were obtained from Hubei Nuona Technology Co. (Wuhan, China) and Sigma-Aldrich (St. Louis, MI, USA), respectively. FLO had a purity of 95% and TFNG had a purity of 99%. TFNG-AM (purity 99%) was synthesized following the protocol laid out by Yang et al. [22]. A gradient-grade acetonitrile for high-performance liquid chromatography (HPLC) analysis was supplied by Honeywell (Burdick and Jackson, Shanghai, China). Sinopharm Chemical Reagent Co. (Shanghai, China) supplied all additional reagents of analytical grade.

Luria-Bertani (LB) medium containing 5 g/L yeast extract, 10 g/L tryptone, and 10 g/L NaCl (pH 7.2) was used to cultivate all *Escherichia coli* strains and *P. salicylatoxidans* CGMCC 1.17248.

### 2.2. Strains and Plasmids

As previously reported [33], our laboratory isolated and preserved *P. salicylatoxidans* CGMCC 1.17248. The protein expression was achieved using Novagen Rosetta (DE3) pLysS cells and a pET28a(+) expression vector from Merck (Kenilworth, New Jersey, USA). *E. coli* Rosetta strains that overexpress *P. salicylatoxidans* CGMCC 1.17248 NHase genes (*anhA* or *anhB*) had already been constructed by our laboratory [33].

### 2.3. Kinetics of FLO Degradation by P. salicylatoxidans CGMCC 1.17248 Resting Cells

*P. salicylatoxidans* CGMCC 1.17248 resting cells were tested for their capacity to degrade FLO. The bacterium was inoculated into 100 mL flasks containing 30 mL of LB medium and incubated for 36 h at 30 °C with shaking at 220 rpm. Following incubation, 3 mL of this culture was inoculated into a 500 mL flask containing 150 mL of LB medium, and a final concentration of 0.1 mmol/L CoCl_2_ was added. The culture was incubated for 24 h in the same conditions. Centrifugation at 8000× *g* for 8 min, followed by two washes with sterilized 50 mmol/L phosphate-buffered saline (PBS, pH 7.5), was used to harvest bacterial cells. After harvesting cells, cell pellets were resuspended in the same buffer containing 0.99 mmol/L FLO, and the optical density of 600 nm was measured and adjusted to 5. HPLC was used to measure residual FLO and its metabolites after samples were collected at the required time intervals.

### 2.4. HPLC and Liquid Chromatography–Tandem Mass Spectrometry (LC–MS)

The concentrations of FLO and its metabolites were measured using a Thermo Scientific UltiMate 3000 (Waltham, USA) Rapid Separation HPLC system outfitted with a Corona charged aerosol detector. The HPLC column and pre-column were, respectively, Agilent reverse-phase HC-C18 columns (4.6 × 250 mm) and reverse-phase C18 pre-columns (4.6 × 20 mm). The mobile phase consisted of a mixture of acetonitrile and deionized water containing 0.01% acetic acid (acetonitrile:water, 30:70 *v*/*v*). At a flow rate of 1 mL/min, the elution was monitored at 265 nm [19]. LC–MS analysis was performed using the method outlined in the Supplementary Materials.

### 2.5. Substrate FLO Testing of AnhA and AnhB

To investigate the FLO transformation ability of *E. coli* pET28a-AnhA and *E. coli* pET28a-AnhB resting cells, as well as of a control *E. coli* strain containing pET28a, bacteria were inoculated into 20 mL of LB medium at 37 °C (220 rpm). Following an initial incubation period of 12 h, 1 mL of this seed culture was added to 100 mL of LB medium, which was then incubated for a further 2.5 h (until the OD_600_ value reached 0.6). Isopropyl β-D-1-thiogalactopyranoside (IPTG) was added to a final concentration of 0.2 mmol/L. After further incubation for 6 h, samples were collected by centrifugation at 8500× *g* for 5 min. The resulting cell sediments were then washed in PBS. The cells were resuspended at OD_600_ = 5 in 5 mL of the PBS buffer with 0.99 mmol/L FLO. Samples were collected after 12 h and centrifuged at 10,000× *g* for 10 min to eliminate any remaining cells. The supernatant was then filtered and diluted to a volume suitable for HPLC analysis of substrates and metabolites.

### 2.6. Cloning of Amidase Genes from P. salicylatoxidans CGMCC 1.17248

The amidases potentially responsible for the transformation of TFNG-AM to TFNG were identified using a protein homology analysis strategy. The genome of *P. salicylatoxidans* CGMCC 1.17248 was sequenced and all annotated proteins were screened as possible amidases. Because the amidases AmiA and AmiB of the N_2_-fixing bacterium *M. flocculans* CGMCC 1.16731 were previously demonstrated to convert TFNG-AM to TFNG, all putative amidase protein sequences were aligned with AmiA and AmiB. A phylogenetic tree was constructed for potential amidases based on sequence similarity using the neighbor-joining algorithm in MEGA 8.0 software (Figure S1). Then, amidase sequences with high homology to the amidases AmiA and AmiB of *M. flocculans* were chosen for gene cloning.

The above-selected amidase-encoding genes were cloned using primers synthesized by Springen Biotechnology Co., Ltd. (Nanjing, China) (Table 1). A Sangon Bacterial Genomic DNA Extraction Kit (Shanghai, China) was used to extract genomic DNA from *P. salicylatoxidans* CGMCC 1.17248. For DNA amplification operations, PrimeSTAR Max DNA Polymerase Premix (Takara, Dalian, China) was employed. The DNAMAN software version 8.0 was used to analyze protein sequence similarity. The PsmiA and PsmiB sequences characterized in the present study have been submitted to GenBank under the indicated accession numbers WHP70861 and WHP70862, respectively.

### 2.7. Gene Expression and Metabolism of Substrate TFNG-AM

Genes encoding amidases were amplified by using the gene-specific primers described in Table 1 and the genomic DNA of *P. salicylatoxidans* CGMCC 1.17248 as the template. After being gel-purified, target fragments were ligated into expression vector pET28a, and recombinant plasmids were transformed into electrocompetent *E. coli* Rosetta (DE3) pLysS cells. The recombinant strains were then induced with IPTG for expression of the amidases.

SDS-PAGE and Coomassie Brilliant Blue R-250 were used to evaluate protein expression. TFNG-AM degradation was achieved using resting cells containing recombinant amidase via the resting cell transformation method as described in the Supplementary Materials.

### 2.8. Half-Life Calculation

Determination of the half-life of FLO was in accordance with the approach described by Zhang et al. [34] (see Supplementary Materials).

### 2.9. Homology Modeling of Amidases

Structural models of amidases PsmiA and PsmiB were constructed based on the crystal structures of *Rhodococcus* sp. N-771 amidase (fold library id: 3a1i.1.A) and *Candida albicans* fatty acid amide hydrolase (fold library id: 6kvr.1.A), respectively. The homology models of these amidases were built and evaluated using the SWISS-MODEL workspace (https://swissmodel.expasy.org/interactive) (accessed on 6 September 2023) and I-TASSER (Iterative Threading ASSEmbly Refinement, http://zhanglab.ccmb.med.umich.edu/I-TASSER/) (accessed on 7 September 2023) [35]. The reliability of the models was assessed using the Global Model Quality Estimation and Quantitative Model Energy Analysis methods [36,37]. PyMOL version 2.4 was employed to further examine the structures and templates of the amidases [38].

### 2.10. Molecular Docking of PsmiA and TFNG-AM

The molecule TFNG-AM was drawn with a 2D structure by using ChemDraw 19.0. The predicted structure of PsmiA was obtained by homology modeling as described in Section 2.9. Using MOE (Molecular Operating Environment) software (version 2022.02 package), the ligand–protein interaction behaviors of TFNG-AM and PsmiA were estimated. Docking was performed by using the Triangle Matcher docking algorithm and London dG scoring function. Calculations of root mean square displacement (RMSD) are critical in comparing different conformers of the same ligand. Docking software generates different ligand conformations, the quality of which is usually assessed by RMSD values [39,40,41]. The 2D interactions of ligands with PsmiA were visualized by the MOE protein–ligand interaction tool.

## 3. Results and Discussion

### 3.1. Degradation of FLO by Resting Cells of P. salicylatoxidans CGMCC 1.17248 and Metabolite Identification

**Figure 1:**
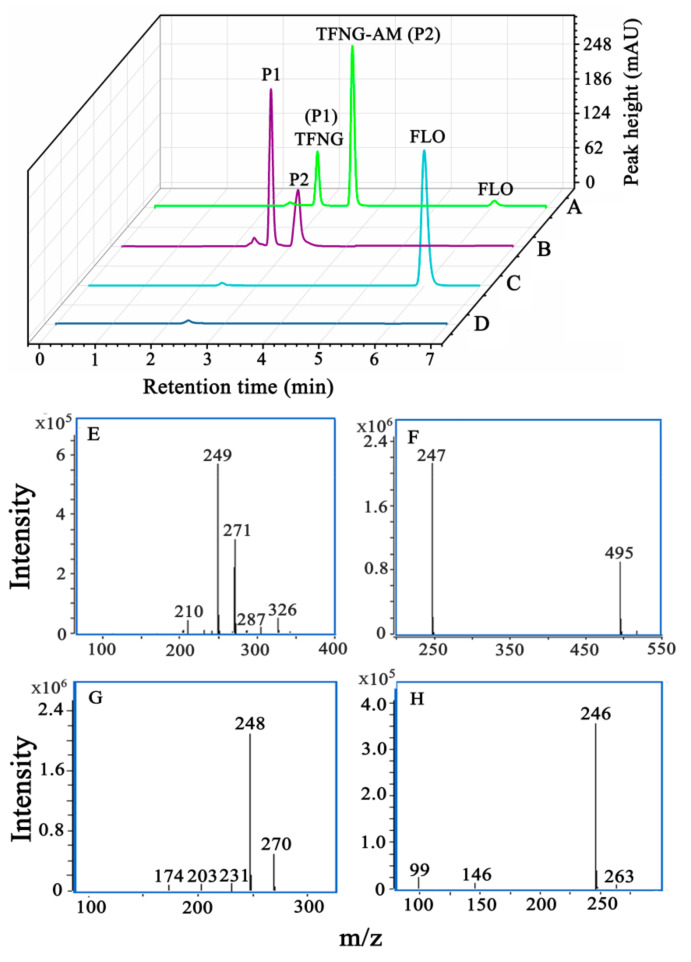
**High-performance liquid chromatography and liquid chromatography–mass spectrometry analysis of flonicamid (FLO) and *N*-(4-trifluoromethylnicotinoyl) glycinamide (TFNG-AM) degradation by resting cells of *Pseudaminobacter* *salicylatoxidans* CGMCC 1.17248.** (**A**) FLO degradation by resting cells of *P. salicylatoxidans* CGMCC 1.17248. (**B**) TFNG-AM degradation by *P. salicylatoxidans* CGMCC 1.17248. (**C**) Substrate-only control (FLO only). (**D**) Bacteria-only control. (**E**) Positive-ion electrospray ionization mass spectrum of metabolite P1. (**F**) Negative-ion electrospray ionization mass spectrum of metabolite P1. (**G**) Positive-ion electrospray ionization mass spectrum of metabolite P2. (**H**) Negative-ion electrospray ionization mass spectrum of metabolite P2.

*P. salicylatoxidans* CGMCC 1.17248 was found to convert FLO into two polar metabolites, as determined by HPLC analysis (Figure 1A,B). The retention times of these metabolites were approximately 2.8 and 3.2 min, which matched the retention times of the standard compounds TFNG and TFNG-AM, respectively, in LC–MS analysis. No detectable peaks corresponding to metabolites of FLO were observed in the substrate or bacterial control samples (Figure 1C,D). Mass spectrometry analysis in positive ion mode revealed that metabolite P1 exhibited peaks at *m*/*z* = 249, 271, and 287, corresponding to [M + H]^+^, [M + Na]^+^, and [M + K]^+^ ions, respectively (Figure 1E,F). Metabolite P2 displayed peaks at *m*/*z* = 248 and 270, corresponding to [M + H]^+^ and [M + Na]^+^ ions (Figure 1G,H). These findings are consistent with the results reported by Zhao et al. [19]. Consequently, metabolites P1 and P2 were identified as TFNG and TFNG-AM, respectively.

Interestingly, the concentration of TFNG-AM increased rapidly during the initial 48 h of incubation, followed by a rapid decrease in concentration. Concurrently, the concentration of TFNG increased. According to the previous study, nitrile hydratase may initially play a catalytic role in the metabolism of FLO to TFNG-AM by *P. salicylatoxidans* CGMCC 1.17248 [19]. With the extension of conversion time, the action of amidases slowly began to take effect, so the concentration of TFNG increased. Based on these results, one or more metabolic enzymes may be involved in the conversion of TFNG-AM to TFNG by *P. salicylatoxidans* CGMCC 1.17248. The findings indicate that *P. salicylatoxidans* CGMCC 1.17248 is capable of metabolizing FLO into TFNG-AM, which is subsequently transformed into TFNG.

### 3.2. Time Course of FLO Degradation by Resting Cells of P. salicylatoxidans CGMCC 1.17248 and Degradation Pathway Analysis

**Figure 2:**
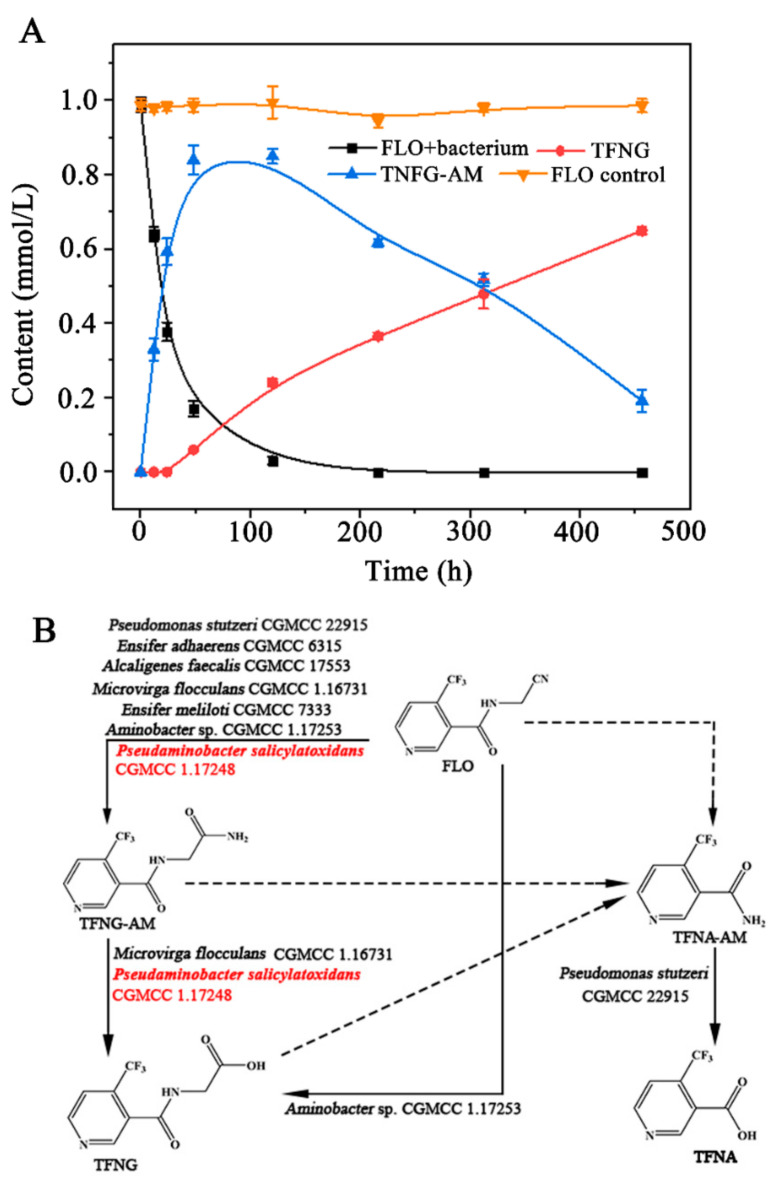
Time course of FLO degradation by resting cells of *P. salicylatoxidans* CGMCC 1.17248 and the proposed metabolic pathway. (**A**) FLO (initial concentration, 0.99 mmol/L) was degraded by resting cells of *P. salicylatoxidans* CGMCC 1.17248. Standard deviations were calculated from triplicate samples from three parallel cultures (*n* = 9). The density (OD_600_) of resting cell suspensions was adjusted to 5. (**B**) The metabolite pathways of FLO in microorganisms.

As shown in Figure 2A, resting cells of *P. salicylatoxidans* CGMCC 1.17248 exhibited a high degradation activity toward FLO, with 61.6% degradation occurring within 24 h. The initial concentration of FLO decreased from 0.99 mmol/L to 0.38 mmol/L during this period. Simultaneously, 0.59 mmol/L of TFNG-AM was generated (a molar conversion rate of 96.7%). The half-life of FLO was found to be 18.7 h. The degradation ability was significantly lower than the values reported for other FLO-degrading bacteria such as the N_2_-fixing *Ensifer adhaerens* CGMCC 6315 (24 h, degradation rate 92%) and *M. flocculans* CGMCC 1.16731 (48 h, degradation rate 94.2%) [19,20], but higher than that of *Variovorax boronicumulans* CGMCC 4969 (48 h, degradation rate 50.9%) [18].

Various species within the genus *Pseudaminobacter* have exhibited proficiency in the degradation of environmental pollutants. For instance, Kim et al. [42] observed that *Pseudaminobacter* sp. SP1a, derived from agricultural soil, contributed to the breakdown of the *N*-methylcarbamate insecticide prothiocarbamate. Zhang et al. [43] proved the capacity of *Pseudaminobacter* sp., isolated from soil suffering from long-term contamination from methyl parathion, to remediate environmental contamination resulting from pesticide usage. The degradation ability of *P. salicylatoxidans* CGMCC 1.17248 suggests its potential as an effective agent for the microbial remediation of FLO.

Nitrile-converting bacteria typically possess nitrilase and NHase/amidase systems. Previous studies revealed that *Alcaligenes faecalis* CGMCC 17553 has a bifunctional nitrilase (named NitA) that transformed FLO into both TFNG and TFNG-AM (Figure 2B). However, no nitrilase gene was identified in the genome of *P. salicylatoxidans* CGMCC 1.17248 or *M. flocculans* CGMCC 1.16731. As a result, amidases are probably involved in the formation of TFNG by these bacteria. *M. flocculans* CGMCC 1.16731 and *V. boronicumulans* CGMCC 4969 could further metabolize the FLO intermediate TFNG-AM to TFNG [18,20]. As a result of these findings, a complete metabolic pathway for FLO degradation can be proposed for *P. salicylatoxidans* CGMCC 1.17248.

This pathway starts with the degradation of FLO into TFNG-AM by enzymes AnhA and AnhB, followed by the hydrolysis of TFNG-AM to TFNG by amidase (Figure 2B).

### 3.3. FLO Degradation by Recombinant Strains

Resting cells of *E. coli* pET28a-AnhA and *E. coli* pET28a-AnhB completely converted FLO to TFNG-AM within 12 h, whereas controls exhibited no degradation activity. Previous studies have shown that AnhA and AnhB exhibited catalytic activity against some neonicotinoid insecticides such as sulfoxaflor and acetamiprid, because nitrile hydratases could hydrolyze the CN group of these nitrile compounds, and the same is true for FLO [33,44]. These results suggest that these two NHases (AnhA and AnhB) are responsible for converting FLO to TFNG-AM. 

### 3.4. Cloning and Overexpression of Amidases in E. coli Rosetta (DE3) and TFNG-AM Degradation by the Recombinant Strains

**Figure 3:**
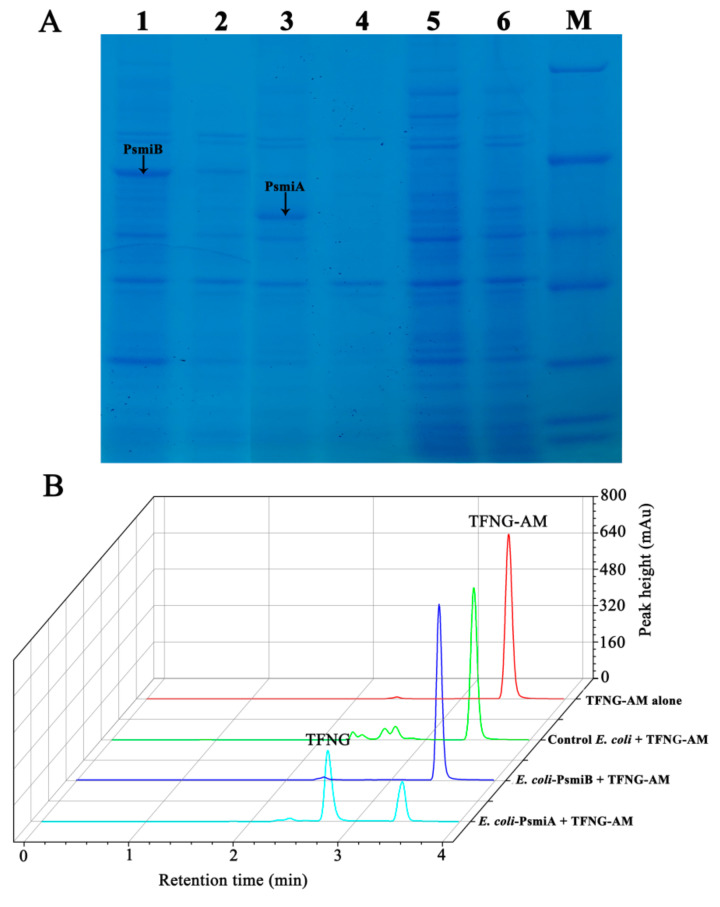
**SDS-PAGE analysis of expression of PsmiA and PsmiB in *Escherichia coli*, and TFNG-AM biodegradation by resting recombinant *E. coli* cells.** (**A**) Lane M, protein molecular weight markers (116, 66.2, 45, 35, 25, 18.4, and 14.4 kDa); Lane 1, total protein from *E. coli* cells expressing PsmiA; Lane 2, soluble protein from *E. coli* cells expressing PsmiA; Lane 3, total protein from *E. coli* cells expressing PsmiB; Lane 4, soluble protein from *E. coli* cells expressing PsmiB; Lane 5, total protein from wild-type *E. coli* (control); Lane 6, soluble protein from wild-type *E. coli* (control). (**B**) Resting cell transformation of TFNG-AM. Substrate control without TFNG-AM; *E. coli* control without TFNG-AM; TFNG-AM degradation by resting cells of *E. coli*-PsmiB; TFNG-AM degradation by resting cells of *E. coli*-PsmiA.

In *E. coli* Rosetta cells, pET28a-based plasmids containing *psmiA* and *psmiB* were successfully introduced, respectively. The expressed target protein PsmiB exhibited good solubility as observed in SDS-PAGE analyses (Figure 3A). However, recombinant PsmiA formed insoluble inclusion bodies, which presumably may result from incorrect folding of the protein. We were not able to improve the soluble expression of PsmiA despite attempts to optimize the conditions. The molecular weights of PsmiA and PsmiB were consistent with their predicted sizes.

The degradation activity of TFNG-AM by the recombinant strains was investigated. Within the first 48 h of incubation, *E. coli*-PsmiA resting cells transformed the available TFNG-AM into TFNG. In contrast, control *E. coli* cells containing the empty pET28a vector and *E. coli*-PsmiB did not exhibit any measurable degradation of TFNG-AM (Figure 3B). These findings strongly suggest that PsmiA is responsible for the metabolic conversion of TFNG-AM to TFNG in *P. salicylatoxidans* CGMCC 1.17248.

### 3.5. Bioinformatic Analyses

The lengths of *psmiA and psmiB* were determined to be 1308 and 1545 base pairs, respectively, corresponding to proteins PsmiA and PsmiB with 435 and 514 amino acid residues, respectively. The G+C contents of the genes were 68.12 mol% and 62.78 mol%, respectively. The molecular weights of PsmiA and PsmiB were predicted to be approximately 45.5 kDa and 55.0 kDa, respectively. The isoelectric points of PsmiA and PsmiB were predicted to be 5.16 and 5.46, respectively. Through protein homology analysis, it was found that these amidases clustered with the amidases AmiA and AmiB from *M. flocculans* CGMCC 1.16731. Further analysis using the Protein Basic Local Alignment Search Tool revealed that PsmiA shared 62.76% and 27.46% amino acid sequence identity with *M. flocculans* CGMCC 1.16731 AmiA and AmiB, respectively. PsmiB showed only 25.82% and 20.54% identity with AmiA and AmiB. Additionally, PsmiA and PsmiB showed 24.62% and 23.67% similarity, respectively, to the amidase PsAmiA from *Pseudomonas stutzeri* CGMCC 22915 (GenBank accession no. UOU03443.1).

**Figure 4:**
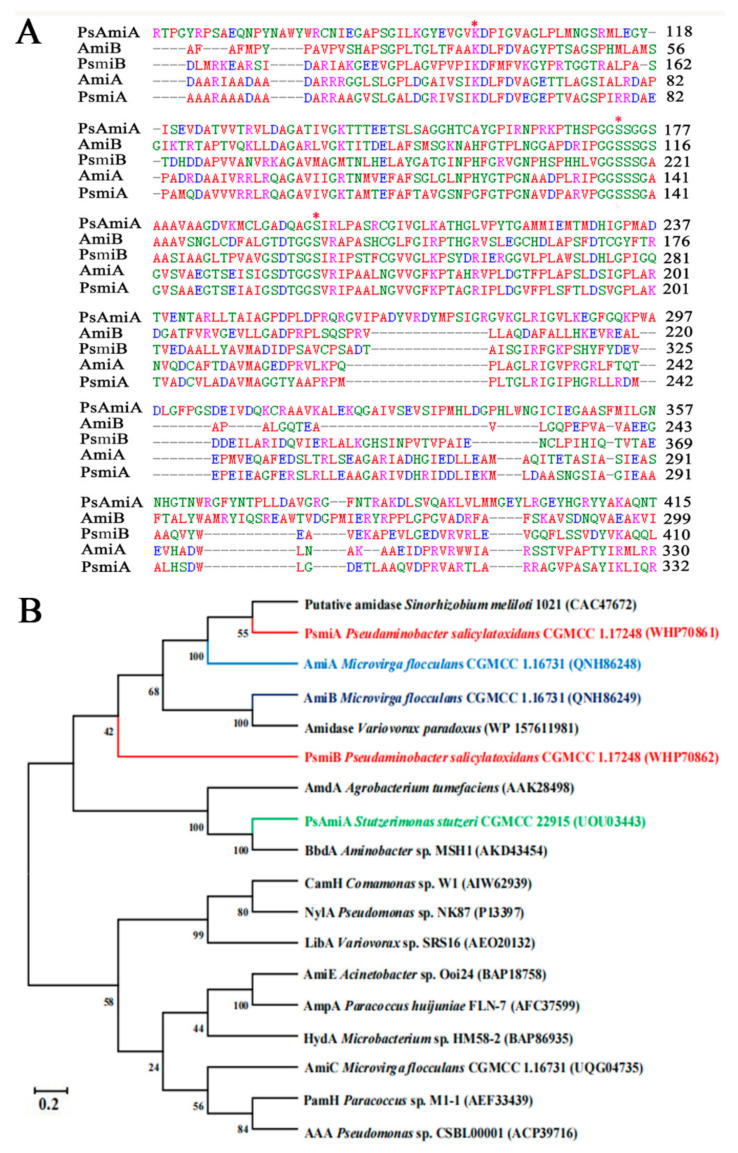
**Alignment of partial amino acid sequences of PsmiA and PsmiB and phylogenetic analysis.** (**A**) Red asterisks indicate residues belonging to the Ser–Ser–Lys catalytic triad. (**B**) Phylogenetic tree showing the relationships between PsmiA, PsmiB, and other amidases from signature family amidases, constructed using the neighbor-joining method. Bootstrap percentages are shown at the nodes from 1000 replicates.

There is a conserved serine- and glycine-rich sequence (GGSSSG) shared by PsmiA and PsmiB, which were identified as members of the amidase family of proteins [27,45]. According to a sequence comparison analysis, both proteins contain the conserved triad of residues Ser161-Ser137-Lys61 and Ser241-Ser217-Lys142, which is characteristic of amidase family members (Figure 4A). Thus, PsmiA and PsmiB belong to the signature amidase family. Based on phylogenetic analysis, PsmiA and PsmiB appear to be closely related to amidases from N_2_-fixing bacteria. Interestingly, however, PsmiB and the two amidases from *M. flocculans* CGMCC 1.16731 (AmiA and AmiB) were located on separate branches of the tree (Figure 4B).

In terms of evolutionary branching, PsmiB is closer to *M. flocculans* CGMCC 1.16731 AmiB (QNH86249) than PsmiA and AmiA, and PsmiB shares only 28.30% amino acid sequence identity with PsmiA. Zhao et al. [19] previously discovered that AmiB could degrade TFNG-AM, but in this investigation, we found that PsmiB does not degrade TFNG-AM, whereas PsmiA does. PsmiA is significantly different from other known signature amidases, with <30% amino acid sequence identity, suggesting that PsmiA is a novel amidase. Interestingly, PsmiA and PsmiB were on different branches of a phylogenetic tree (Figure S1), indicating evolutionary divergence between them. Recently, Jiang et al. [21] identified an amidase (UOU03443) in *S. stutzeri* CGMCC 22915 that catalyzes the formation of TFNA-AM from FLO, another intermediate of FLO. However, it is unable to catalyze the conversion of TFNG and shares only 24.71% amino acid sequence similarity with PsmiA. This finding suggests that amidases belonging to the same family may exhibit strict substrate specificity.

### 3.6. Predicted Structures of PsmiA and PsmiB

**Figure 5:**
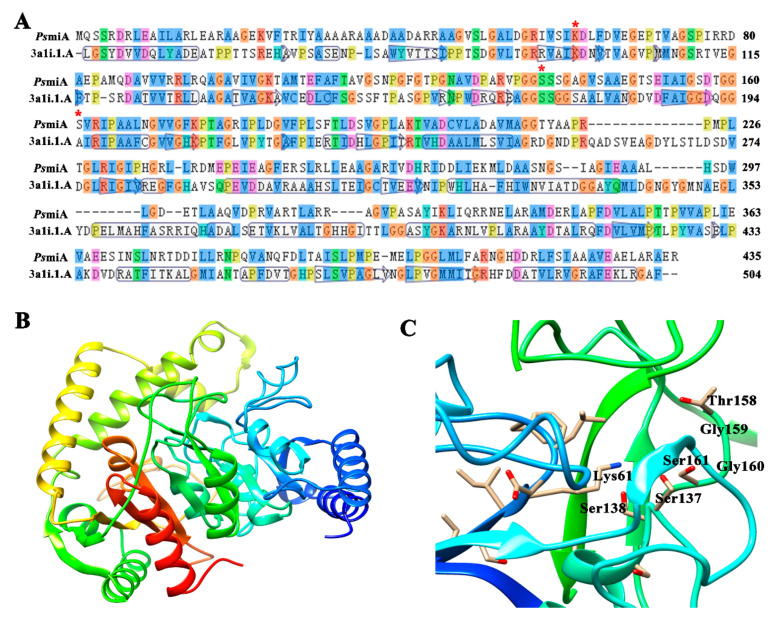
**Homology modeling of PsmiA.** (**A**) Sequence alignment of PsmiA and the selected amidase template, and analysis of key residues. (**B**) Homology model of the three-dimensional structure of PsmiA. (**C**) The predicted structure of PsmiA contains the Ser161–Ser137–Lys61 catalytic motif and other important active site residues. * indicated the amino acid residues of the catalytic triad.

Figure 5A and Figure S2A show that PsmiA and PsmiB have 27.27% and 31.18% amino acid sequence similarity to *Rhodococcus* sp. N-771 amidase and *C. albicans* fatty acid amide hydrolase, respectively. As illustrated in Figure 5B, PsmiA was predicted by homology modelling to consist of 19 helices and 10 strands. There is a predicted catalytic site, within the substrate-binding pocket, that contains the Ser16–Ser137–Lys61 catalytic motif. Active site residues Ser138, Thr158, Gly159, and Gly160 are also present in the catalytic pocket (Figure 5C). As predicted (Figure S2B), PsmiB consists of 20 helices and nine strands. Ser241–Ser217–Lys142, the catalytic motif, is incorporated into the predicted substrate-binding cleft, while active site residues Ser218, Thr238, and Gly240 are positioned at a distance from the catalytic pocket (Figure S2C).

There may be a spatial difference between major active sites and the catalytic pocket that affects substrate binding efficiency, thus explaining the observed variation in PsmiA and PsmB activity. Previous studies have confirmed that when the catalytic triad of PsAmiA (residues K98A, S173A, and S197) was mutated, the protein still showed good solubility but it lost TFNA-AM degradation activity. These results indicate that these specific amino acid residues are crucial for the degradation of the substrate [21].

Among signature-type amidases, Asp191 and Ser195 are highly conserved active site residues, and substitution of these residues completely inhibits enzyme activity in *Rhodococcus* [30]. Interestingly, PsmiA has a phenylalanine residue at position 191 instead of asparagine, while PsmiB contains a threonine at position 195 in place of serine. We hypothesize that these substitutions of these crucial residues may impede the catalytic activity of the amidases toward TFNG-AM; this warrants further investigation.

### 3.7. Structure–Function Relationship of PsmiA

**Figure 6:**
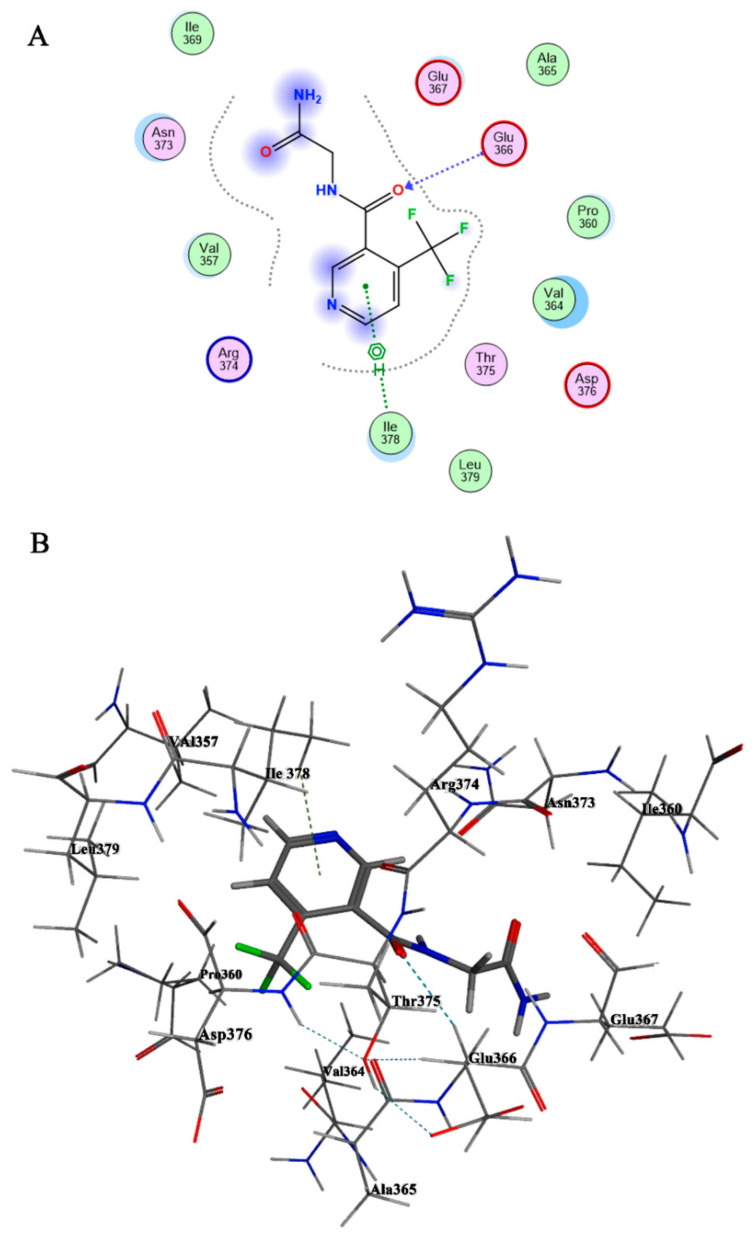
**2D (A) and 3D (B) binding modes of TFNG-AM in the active site of PsmiA.** The active site residues of PsmiA are presented in the model. The ligands in the active site of PsmiA are shown as sticks, and hydrogen bonds are represented by dashed lines.

Calculations from molecular docking experiments of the structural model TFNG-AM of PsmiA with ligand TFNG-AM are shown in Table S1. The list has been ordered by how well the ligand binds, with the docking score and the “S” column serving as the primary reference criteria: a lower value indicates that the ligand is more strongly bound to the receptor. Compound TFNG-AM exhibited the highest binding affinity and RMSD (value 3.3902 Å) to PsmiA, as summarized in Table S1. The S value for it was −5.0515, and it strongly interacted with the binding site by forming two H-bonds, with residues Glu247 and Met242 (Figure 6A). In the catalytic pocket, the pyridine ring and trifluoromethyl group of TFNG-AM formed hydrophobic interactions with hydrophobic amino acids Leu239, Pro244, Pro356, and Ile378 (Figure 6A). These hydrophobic interactions stabilized the conformation of TFNG-AM in the catalytic pocket and ensured the nucleophilic attack of Ser161 on TFNG-AM. Based on the different interaction modes of the ligand with the hydrophilic amino acid backbone in the PsmiA binding site (Figure 6B), we postulate that the hydrophilic fragments in residues Glu243, Glu247, Arg240, and Asp241 play important roles in the binding of molecules to PsmiA.

## 4. Conclusions

This study provides evidence that *P. salicylatoxidans* CGMCC 1.17248 possesses the ability to effectively break down the systemic insecticide FLO into TFNG-AM and TFNG using an NHase/amidase pathway. A previously unknown amidase, which belongs to a unique signature family, has been identified as playing a role in the degradation of TFNG-AM. PsmiA holds promise for applications in environmental remediation and the synthesis of novel amides. The predicted key amino acids may have an impact on the activity of amidase, and this study provides a perspective for understanding the structure–function relationship of amidase and the microbial remediation of FLO.

## Figures and Tables

**Table 1:** Primers used in this study.

Target	Primer	Sequence (5’→3’) ^a^	Amplicon Size (bp)
*psmiA*	F1	ACAGCAAATGGGTCGCGGATCCGAATTCATGCAGTCCAGCCGCGAT	1308
	R1	ATCTCAGTGGTGGTGGTGGTGGTGCTCGAGTCAACGCTCCGCCCTGGC	
*psmiB*	F2	ACAGCAAATGGGTCGCGGATCCGAATTCATGCCGACGATGATCGATGC	1545
	R2	ATCTCAGTGGTGGTGGTGGTGGTGCTCGAGTCAAAGTTCAGAGATGACTGCCTG	

^a^ Underlined bases indicate restriction enzyme cleavage sites for *Eco*RI (GAATTC) and *Xho*I (CTCGAG).

## Data Availability

The original contributions presented in the study are included in the article/Supplementary Materials, further inquiries can be directed to the corresponding authors.

## Supplementary Materials

The following supporting information can be downloaded at: https://www.mdpi.com/article/10.3390/microorganisms12061063/s1. Liquid chromatography-mass spectrometry (LC-MS) analyses; Resting cell transformation method; Half-life of FLO; Table S1: Simulated molecular docking energies (kcal/mol) of the tested ligands; Figure S1: Phylogenetic tree showing the relationships among amidases from *P. salicylatoxidans* CGMCC 1.17248; Figure S2: Homology modeling analysis of PsmiB.
