# Supplementary material for: Efficient Biodegradation of the Neonicotinoid Insecticide Flonicamid by Pseudaminobacter salicylatoxidans CGMCC 1.17248: Kinetics, Pathways, and Enzyme Properties
Source: Microorganisms. 2024 May 24;12(6):1063. doi: 10.3390/microorganisms12061063 (PMC11205548; doi:10.3390/microorganisms12061063)

Article

Efficient Biodegradation of the Neonicotinoid Insecticide Flonicamid by *Pseudaminobacter salicylatoxidans* CGMCC 1.17248: Kinetics, Pathways, and Enzyme Properties

Yun-Xiu Zhao [1,†], Jing Yuan [2,†], Ke-Wei Song [1], Chi-Jie Yin [1], Li-Wen Chen [3], Kun-Yan Yang [3], Ju Yang [3,*] and Yi-Jun Dai [2,*]

[1] Jiangsu Key Laboratory for Bioresources of Saline Soils, Jiangsu Synthetic Innovation Center for Coastal Bio-Agriculture, School of Wetlands, Yancheng Teachers University, Yancheng 224007, China; zhaoyx@yctu.edu.cn (Y.-X.Z.); 13773187821@163.com (K.-W.S.); yinchijie@outlook.com (C.-J.Y.)

[2] Jiangsu Key Laboratory for Microbes and Functional Genomics, Jiangsu Engineering and Technology Research Center for Industrialization of Microbial Resources, College of Life Science, Nanjing Normal University, Nanjing 210023, China; 231201019@njnu.edu.cn

[3] College of Marine and Biological Engineering, Yancheng Teachers University, Yancheng 224002, China; clw2212410431@163.com (L.-W.C.); carrie041029@outlook.com (K.-Y.Y.)

* Correspondence: yangj01@yctu.edu.cn (J.Y.); daiyijun@njnu.edu.cn (Y.-J.D.); Tel.: +86-25-85891731 (Y.-J.D.)

† These authors contributed equally to this work.

Liquid chromatography-mass spectrometry (LC-MS) analyses

Metabolites were identified by LC-tandem mass spectrometry (LC-MS/MS) analysis using an Agilent LC-MS system (model 1290 Infinity LC/6460 Triple Quad MS; Agilent Technologies, Santa Clara, CA) equipped with an Agilent Jet Stream electrospray ionization source. The column was the same as that in HPLC analysis and the flow-rate was 0.6 mL/min. Data acquisition was performed in positive and negative-ionization mode, respectively.

Resting cell transformation method

Bacteria were inoculated into LB medium and incubated at 37 °C on a shaker for 12 h. When the cell density (OD_{600}) reached ~0.6, 2% volume of IPTG (0.1 mol/L) was added to the medium. After continuing the incubation at 30 °C and 220 rpm for 5 h, the bacterial cells were collected by centrifugation. The harvested cells were washed twice with phosphate-buffered saline (PBS; 50 mmol/L, pH 7.0). Then, 5 mL of 200 mg/L TFNG-AM dissolved in PBS was

added to the *E. coli* cells (OD_{600} = 2.5). The mixture was shaken at 30 °C, 220 rpm, and sampled every 10 min. Subsequently, the supernatant was centrifuged at 9000 × *g* for 10 min, filtered, and analyzed by HPLC.

Half-life of FLO

Half-life value was determined by plotting $\ln(I/I_0)$ against time using the equation $\ln(I/I_0) = -kt$, where I_0 and I represent the initial and residual concentrations, respectively. The half-life was calculated as $t_{1/2} = (\ln 2)/k$, where $t_{1/2}$ is the half-life and k is the apparent elimination constant. The first-order equation provided a satisfactory fit for the data ($r > 0.9$).

Table S1 Simulated molecular docking energies (kcal/mol) of the tested ligands

	S	rsmd_refine	E_conf	E_place	E_score1	E_refine
**TFNG-AM**	-5.0515	3.3902	-27.9165	-36.6175	-5.6566	-25.9004
**TFNG-AM**	-4.9976	0.8818	-25.9022	-47.2123	-5.9008	-13.3159
**TFNG-AM**	-4.6580	1.4873	-27.4534	-39.7078	-6.6875	-22.3342
**TFNG-AM**	-4.5929	1.5337	-28.8351	-40.2665	-6.4882	-21.6519
**TFNG-AM**	-4.4953	2.2473	-27.3379	-45.9404	-7.1882	-17.4249

S: The final score, which is the score of the last stage that was not set to None; rmsd_refine: The root mean square deviation between the pose before refinement and the pose after refinement; E_conf: binding energy for the ligand-conformer; E_place: binding energy for the ligand-receptor; E_score1: Score from rescoring stages 1 and 2; E_refine: Score from the refinement stage, calculated to be the sum of the van der Waals, electrostatic and solvation energies, under the generalized Born solvation model (GB/VI).

Figure S1 Phylogenetic tree showing the relationships among amidases from *P. salicylatoxidans* CGMCC 1.17248

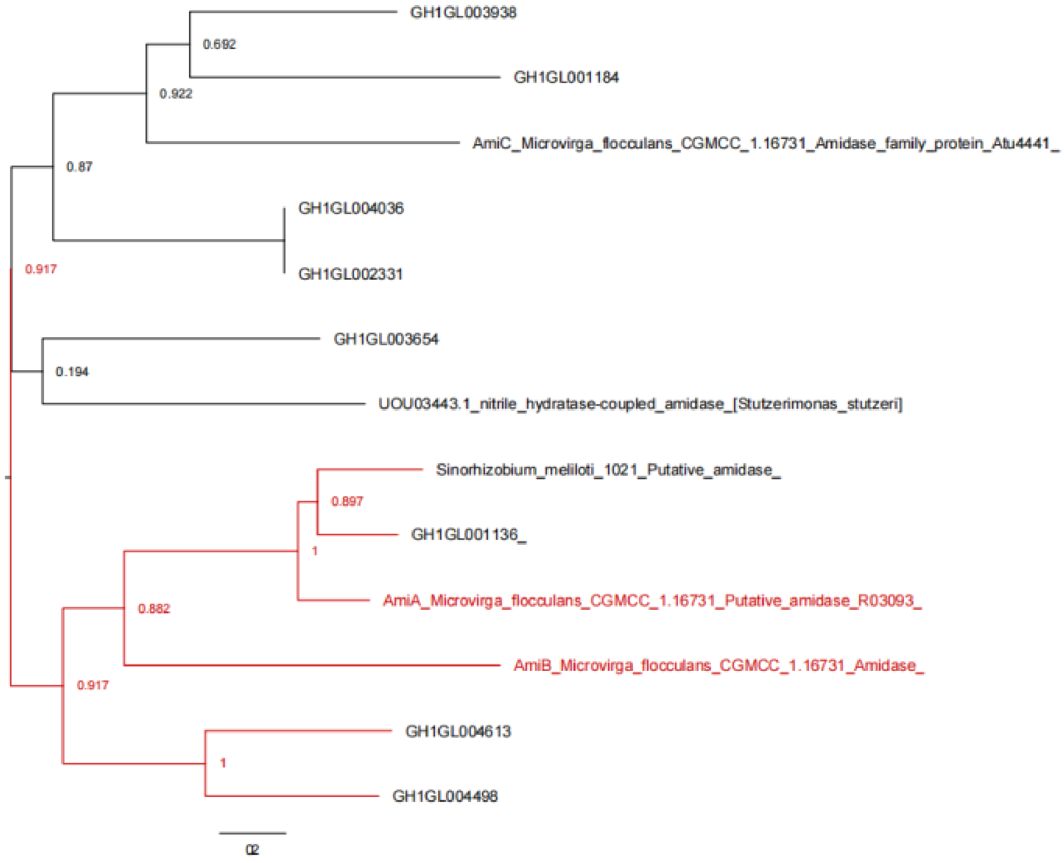

The scale bar indicates 0.2 substitutions per nucleotide position.

Figure S2 Homology modeling analysis of PsmiB

(A) Sequence alignment of PsmiB and the selected amidase template and analysis of the key sites. (B) Homology model of the three-dimensional structure of PsmiB. (C) The predicted structure of PsmiB contains the Ser241-Ser217-Lys142 catalytic motif and other active site residues.

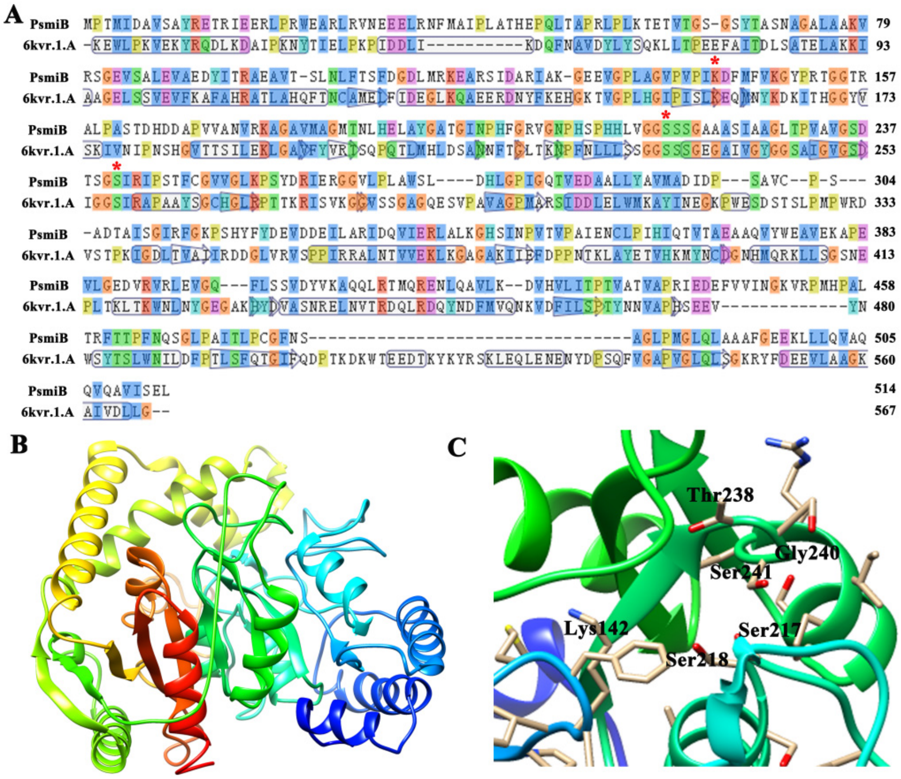

Supplement: Supplementary file 1 [file microorganisms-12-01063-s001.zip › microorganisms-2987294-supplementary.pdf]
